# Flaxseed oil intake reduces serum small dense low-density lipoprotein concentrations in Japanese men: a randomized, double blind, crossover study

**DOI:** 10.1186/s12937-015-0023-2

**Published:** 2015-04-21

**Authors:** Yuka Kawakami, Hisami Yamanaka-Okumura, Yuko Naniwa-Kuroki, Masae Sakuma, Yutaka Taketani, Eiji Takeda

**Affiliations:** Department of Clinical Nutrition and Food Management, Institute of Health Biosciences, University of Tokushima Graduate School, 3-18-15 Kuramoto, Tokushima, 770-8503 Japan

**Keywords:** Alpha-linolenic acid, n-3 fatty acids, Small dense LDL, Triglyceride, Human study

## Abstract

**Background:**

The effects of alpha-linolenic acid (ALA) on cardiovascular risk factors considerably vary between published reports. Therefore, we investigated the effects of 12-week supplementation with flaxseed oil (FO), which is a rich source of ALA, on cardiovascular risk factors such as serum small dense low-density lipoprotein (sd-LDL) concentrations.

**Methods:**

In a randomized, double blind, crossover study, 15 subjects ingested 10 g of FO or corn oil (CO), containing 5.49 g and 0.09 g of ALA, respectively, once daily with dinner. Blood samples were collected at 0, 4 and 12 weeks, and were used for analysis of serum lipid, lipid-related proteins, serum fatty acids and serum sd-LDL cholesterol. Differences during the test period were identified using a repeated-measures analysis of variance (ANOVA) for within-group effects. Group differences were identified using paired t-test at each blood sampling time point.

**Results:**

ALA and eicosapentaenoic acid concentrations were significantly higher in the FO period at 4 and 12 weeks than in the CO period. No significant differences in docosahexaenoic acid concentrations were observed between two periods, and cholesteryl ester transfer protein and apolipoprotein B concentrations were significantly lower in the FO period than in the CO period at 12 weeks. FO supplementation was associated with a significant decrease in sd-LDL concentrations at 4 and 12 weeks, and CO supplementation had no effect. Moreover, sd-LDL concentrations were significantly lower in the FO period than in the CO period at 4 weeks. Among subjects with triglyceride (TG) concentrations of >100 mg/dl, FO supplementation markedly reduced sd-LDL concentrations at 4 and 12 weeks compared with baseline. Sd-LDL concentrations significantly differed between the periods at both 4 and 12 weeks.

**Conclusion:**

This study indicates that the FO, which is a rich source of ALA, leads to lower sd-LDL cholesterol concentrations.

## Background

Metabolic syndrome is a cluster of inter-related plasma lipid and lipoprotein abnormalities, including reduced high-density lipoprotein (HDL) cholesterol, a predominance of small dense low-density lipoprotein (sd-LDL) particles, and increased triglyceride (TG) concentration [1]. These dyslipidemic features are associated with increased risk of cardiovascular disease (CVD) [2].

Subclasses of LDL are characterised by variations in density, size and chemical composition and are of clinical importance [3]. Sd-LDL is a risk factor for the development of coronary artery disease in Westerners [4,5] and Japanese with relatively low LDL cholesterol concentrations [6,7]. In a recent study, sd-LDL cholesterol was significantly associated with CVD development in a Japanese population [8], indicating the importance of sd-LDL cholesterol as a biomarker to predict CVD.

Numerous studies have investigated the influence of n-3 fatty acids on CVD risk, and most of the health benefits observed in these studies have been attributed to the seafood n-3 fatty acids, eicosapentaenoic acid (EPA) and docosahexaenoic acid (DHA) [9,10]. Compared with seafood n-3 fatty acid, fewer studies have evaluated the relationship between plant-derived n-3 fatty acid, alpha-linolenic acid (ALA), and the risk of coronary heart disease and other CVD outcomes. Previous meta-analysis showed that consumption of ALA might reduce heart disease mortality [11], whereas other previous study showed no significant cardiovascular benefit of ALA supplementation [12,13]. Thus, the effects of ALA have been inconsistent, and previous systematic review pointed out some methodologic problems [12]. However, recently there has been a steady increase in the number of studies supporting specific health benefits of ALA [14,15]. Furthermore, because seafood n-3 fatty acids consumption is limited by availability, plant-based n-3 fatty acids containing ALA, which can be elongated and desaturated to EPA and DHA [16], may be an important dietary alternative source for the maintenance of optimal EPA and DHA concentrations in plasma and cell membranes. Therefore, characterization of the cardioprotective effects of ALA is of considerable importance with regard to public health.

Therefore, the objective of this study was to elucidate the effects of 12-week supplementation with flaxseed oil (FO), which is a rich source of ALA, on cardiovascular risk factors, including sd-LDL cholesterol.

## Methods

### Subjects

Twenty-six adult males with healthy and social lifestyles were screened. Potential participants were screened by medical history, physical examination, laboratory analysis, and daily intake of fish. Subjects with evidence of infections, diabetes mellitus, renal, liver or inflammatory disease were excluded. Subjects who regularly consumed FO or perilla oil were also excluded. Among twenty-one enrolled men, 1 withdrew before completion of the study due to medication and 5 subjects were excluded for non-compliance during the 12-week intervention. From these participants, 15 subjects with a mean ± standard error (SE) body mass index (BMI, kg/m^2^) of 25.1 ± 0.5 and a mean ± SE age of 44.5 ± 3.1 y completed the study. Four subjects were smokers and 5 had received anti-hypertensive drugs. Subject characteristics and laboratory data are presented in Table 1. According to TG concentrations, 15 subjects were divided into two groups: the TG < 100 mg/dl group (n = 5), and the TG > 100 mg/dl group (n = 10).

**Table 1:** **Characteristics of the subjects (Mean values with their standard errors, 15 males)**

		All (n = 15)	TG < 100 mg/dl (n = 5)	TG > 100 mg/dl (n = 10)
Age	(year)	44.5 ± 3.1	40.2 ± 5.3	46.7 ± 3.9
Body weight	(kg)	71.2 ± 2.3	68.3 ± 4.1	72.7 ± 2.8
BMI	(kg/m^2^)	25.1 ± 0.5	24.8 ± 0.8	25.2 ± 0.6
Abdominal circumference	(cm)	88.0 ± 1.6	85.0 ± 2.6	89.5 ± 1.9
Systolic blood pressure	(mmHg)	135.4 ± 3.8	128.2 ± 4.6	139.0 ± 4.9
Diastolic blood pressure	(mmHg)	77.8 ± 2.9	70.8 ± 3.3	81.3 ± 3.7
Total cholesterol	(mg/dl)	212.6 ± 6.2	220.8 ± 10.9	208.5 ± 7.6
LDL- cholesterol	(mg/dl)	132.7 ± 6.1	146.0 ± 9.5	126.1 ± 7.2
HDL- cholesterol	(mg/dl)	55.2 ± 2.5	57.6 ± 2.9	54.0 ± 3.5
non HDL- cholesterol	(mg/dl)	157.4 ± 6.8	163.2 ± 11.9	154.5 ± 8.7
Small dense LDL-cholesterol	(mg/dl)	41.1 ± 5.2	30.6 ± 9.5	46.3 ± 5.8
Triglyceride	(mg/dl)	124.5 ± 11.8	79.8 ± 3.7	146.8 ± 12.5*
n-6/n-3 ratio		5.4 ± 0.6	5.6 ± 1.0	5.3 ± 0.7
Fasting plasma glucose	(mg/dl)	104.1 ± 2.5	106.8 ± 3.7	102.7 ± 3.3
HbA1c (NGSP)	(%)	5.4 ± 0.1	5.5 ± 0.1	5.4 ± 0.1
Fasting serum insulin	(μU/ml)	7.9 ± 1.2	6.9 ± 2.1	8.4 ± 1.4
Aspartate aminotransferase	(U/l)	21.3 ± 1.3	20.8 ± 1.9	21.5 ± 1.8
Alanine aminotransferase	(U/l)	24.7 ± 2.3	24.0 ± 3.0	25.1 ± 3.1
γ-glutamyl transpeptidase	(U/l)	41.7 ± 7.2	38.0 ± 8.0	43.6 ± 10.3
Creatinine	(mg/dl)	0.8 ± 0.03	0.8 ± 0.04	0.8 ± 0.04
Urea nitrogen	(mg/dl)	12.4 ± 0.4	11.7 ± 1.1	12.8 ± 0.4

Values are mean ± SE.

*Mean value was significantly different from that for TG < 100 mg/dl; p <0.05, Mann–Whitney U test.

Informed consent was obtained from all participants before the start of the study, which was approved by the ethical committee of Tokushima University Hospital, Tokushima, Japan. The study was performed in accordance with the Helsinki Declaration.

### Study design

In a randomized, double blind, crossover study, subjects were given either FO or corn oil (CO) during two consecutive 12-week supplementation periods. The supplementation periods were separated by a 8-week wash-out period. The subjects were instructed to ingest 10 g of FO or CO using the spoon provided, once daily with dinner. Supplements of 10 g of FO and CO contained 5.49 and 0.09 g of ALA, respectively. Fatty acid compositions of the two oils are presented in Table 2. The fatty acid composition of the two oils was measured by gas–liquid chromatography. To avoid oxidation, they were instructed to put oil bottles in a box for blocking out light and to store in the refrigerator.

**Table 2:** **Fatty acid composition of corn oil and flaxseed oil**

		Corn oil	Flaxseed oil
Fatty acid		g/100 g	
14:0	Myristic acid	0.1	0.1
16:0	Palmitic acid	12.0	5.2
16:1	Palmitoleic acid	0.2	0.1
17:0	Heptadecanoic acid	0.1	0.1
18:0	Stearic acid	2.0	3.8
18:1	Oleic acid	29.1	18.9
18:2n-6	Linoleic acid	54.5	16.2
18:3n-3	Alpha linolenic acid	0.9	54.9
20:0	Arachidic acid	0.5	0.2
20:1	Eicosenoic acid	0.3	0.2
20:2n-6	Eicosadienoic acid	0.0	0.1
22:0	Behenic acid	0.1	0.2
24:0	Lignoceric acid	0.2	0.0
n-6/n-3 ratio		60.6	0.3

Subjects were instructed to maintain their habitual diet throughout the study to exclude influences of dietary nutrients, and were asked to continue with their normal daily activities. Subjects were also instructed to avoid intake of anti-inflammatory drugs, vitamins or other dietary supplements throughout the intervention period.

Subjects recorded their daily activities and food intake in a diary, and these life-style records were confirmed during interviews every 2 weeks. Subjects returned used oil bottles and were provided with oil supplements for the following 2 weeks. Remaining volumes in returned bottles were measured to assess compliance. All subjects consumed more than 97% of the assigned oil quantity.

Subjects were visited at 0 (run-in period), 4 and 12 weeks to collect fasting blood samples, body weight, and blood pressure measurement, and to hand in their previous 3-day’s dietary records. Subjects were instructed to eat and drink the same prescribed foods at 20.00 h prior to scheduled visits. Fasting blood samples were collected at 08.00 h after overnight fasting, and were used for analysis of serum lipid, lipid-related proteins, serum fatty acids and serum sd-LDL cholesterol. A dietician calculated mean energy intake from each subject’s dietary records of the 3 days leading up to scheduled visits. These dietary records were analyzed using computerized software (Excel Eiyou-kun version 4.0, Kenpaku-sha, Tokyo, Japan) to determine caloric intake and macronutrient content.

### Analytic methods

Blood sample were centrifuged at 3000 rpm for 10 min at 4°C and then separated into plasma or serum. Alpha-tocopherol was measured using high-performance liquid chromatography. Total cholesterol was determined by the cholesterol dehydrogenase (UV-End) method. LDL-cholesterol and HDL-cholesterol were determined by a direct method. We calculated non-HDL cholesterol as the difference between total and HDL cholesterol. TG was determined by an enzyme method. The remnant like particles (RLP) -cholesterol was determined by the immune adherence method. Cholesterol ester transfer protein activity (CETP) was measured using a commercially available ELISA kit (Daiichi-kagaku, Tokyo Japan). Apolipoproteins (apo A-1, B, C-3 and E) were measured by the turbidimetric immunoassay (TIA) system. Apo B-48 was measured by the chemiluminescent enzyme immunoassay (CLEIA). Serum fatty acids composition was measured by gas–liquid chromatography. Briefly, total lipids in the serum were extracted using the Folch procedure and fatty acids were then methylated with BF_3_/methanol. Transesterified fatty acids was then analyzed using a gas chromatograph (GC-17A; Shimadzu, Kyoto, Japan) with a capillary column Omegawax 250 (Supelco, Bellefonte, PA). Measurements of sd-LDL-cholesterol were performed using sd-LDL-EX “SEIKEN” (Denka Seiken, Tokyo, Japan).

### Statistical analysis

Data are presented as the mean ± SE. Baseline physical characteristic and laboratory data between the TG > 100 mg/dl group and the TG < 100 mg/dl group were compared using Mann–Whitney U test. Differences during the test period were identified using a repeated-measures analysis of variance (ANOVA) and the Bonferroni method for calculating 95% confidence interval (CI) after correcting for multiple comparisons for within-group effects. Group differences were identified using paired t-test at each blood sampling time point. Differences were considered significant when P < 0.05. Statistical analyzes were performed using SPSS for Windows, release 19.0 (SPSS, Chicago, IL).

## Results

### Food and nutrient intake

Dietary intake in CO and FO periods is shown in Table 3. At 0 week, no significant differences in total energy, protein, carbohydrate, fat, dietary fiber or alcohol intake were observed between the periods.

**Table 3:** **Dietary intakes of foods and nutrients in corn oil and flaxseed oil periods**

		Corn oil period			Flaxseed oil period		
		0 week	4 week	12 week	0 week	4 week	12 week
Energy	(kcal)	2054.3 ± 56.5	2266.3 ± 73.3	2289.8 ± 76.2^*^	2146.3 ± 105.8	2349.2 ± 118.6	2261.4 ± 78.0
Protein	(g)	69.2 ± 3.2	74.0 ± 3.4	76.3 ± 2.0	74.3 ± 5.2	75.6 ± 4.5	73.6 ± 3.6
Carbohydrate	(g)	291.2 ± 11.2	304.3 ± 12.9	302.5 ± 15.3	311.1 ± 16.7	315.5 ± 17.8	315.2 ± 15.9
Fat	(g)	51.9 ± 2.6	67.8 ± 3.7^*^	71.4 ± 3.2^*^	54.5 ± 4.8	70.8 ± 4.2^*^	64.1 ± 4.1
alpha-linolenic acid	(mg)	925.0 ± 105.0	1929.0 ± 559.0	1606.0 ± 136.0	1082.0 ± 161.0	6496.0 ± 216.0^*†^	6032.0 ± 140.0^*†^
Eicosapentaenoic acid	(mg)	250.0 ± 71.0	158.0 ± 48.0	153.0 ± 59.0	269.0 ± 83.0	153.0 ± 55.0	225.0 ± 75.0
Docosahexaenoic acid	(mg)	484.0 ± 125.0	338.0 ± 76.0	288.0 ± 85.0	498.0 ± 140.0	329.0 ± 97.0	383.0 ± 109.0
Linoleic acid	(mg)	6410.0 ± 592.0	13633.0 ± 927.0^*^	15675.0 ± 688.0^*^	7902.0 ± 850.0	10623.0 ± 1182.0	8106.0 ± 775.0^†^
n-6/n-3 ratio		4.7 ± 0.7	7.7 ± 0.7^*^	8.3 ± 0.8^*^	5.7 ± 0.9	1.5 ± 0.1^*†^	1.2 ± 0.1^*†^
Cholesterol	(mg)	289.1 ± 31.0	298.7 ± 37.9	344.8 ± 23.8	296.4 ± 40.3	319.7 ± 40.3	306.9 ± 44.2
Dietary fiber	(g)	8.3 ± 0.9	9.9 ± 0.6	10.7 ± 0.6^*^	8.5 ± 0.8	9.8 ± 0.7	10.5 ± 1.1
alpha-tocopherol	(mg)	4.4 ± 0.4	6.7 ± 0.5^*^	8.1 ± 0.5^*^	4.9 ± 0.7	5.9 ± 0.6	5.4 ± 0.5^†^
Alcohol	(g)	16.5 ± 5.9	18.2 ± 5.4	17.0 ± 8.2	16.2 ± 6.6	20.1 ± 7.8	15.8 ± 5.4

Values are means ± SE, n = 15.

*; p <0.05 vs. baseline, repeated-measures ANOVA with Bonferroni correction, †; p <0.05 vs. corn oil, paired t test.

In the FO period, total ALA intake was significantly higher at 4 and 12 weeks than at 0 week. Total ALA intake at 4 and 12 weeks was significantly higher in the FO period than in the CO period. In the CO period, total linoleic acid (LA) intake was significantly higher at 4 and 12 weeks than at 0 week. Total LA intake at 12 weeks was significantly lower in the FO period than in the CO period.

In the FO period, ratios of n-6/n-3 were significantly lower at 4 and 12 weeks than at 0 week. In the CO period, ratios of n-6/n-3 were significantly higher at 4 and 12 weeks than at 0 week. Ratios of n-6/n-3 at 4 and 12 weeks were significantly lower in the FO period than in the CO period.

Alpha-tocopherol intakes at 12 weeks were significantly lower in the FO period than in the CO period.

### Anthropometric and laboratory data

Anthropometric and laboratory parameters from subjects of CO and FO periods are shown in Table 4. No significant differences in body weight were observed at 0 week, and no significant differences in alpha-tocopherol concentrations were observed between the two periods at the completion of the study.

**Table 4:** **Anthropometric and laboratory parameters in corn oil and flaxseed oil periods**

		Corn oil period			Flaxseed oil period		
		0 week	4 week	12 week	0 week	4 week	12 week
Body weight	(kg)	72.2 ± 2.5	72.0 ± 2.5	72.4 ± 2.6	71.4 ± 2.4	71.9 ± 2.4	72.2 ± 2.5
Systolic blood pressure	(mmHg)	132.4 ± 3.0	132.6 ± 3.3	126.1 ± 2.6	132.9 ± 4.3	134.8 ± 3.9	129.7 ± 3.5
Diastolic blood pressure	(mmHg)	76.4 ± 2.6	77.0 ± 2.2	74.8 ± 2.4	77.6 ± 2.9	81.9 ± 3.2^†^	75.2 ± 3.0
Fasting plasma glucose	(mg/dl)	102.5 ± 2.5	99.9 ± 2.5	102.5 ± 2.2	101.9 ± 2.2	102.2 ± 1.8	102.1 ± 1.9
HbA1c	(%)	4.9 ± 0.1	4.9 ± 0.1	4.9 ± 0.1	5.0 ± 0.1	4.9 ± 0.1	5.0 ± 0.1
Fasting serum insulin	(μU/ml)	8.5 ± 1.1	9.5 ± 1.4	9.1 ± 1.3	8.3 ± 1.3	9.1 ± 1.1	9.4 ± 1.5
Aspartate aminotransferase	(U/l)	22.5 ± 2.0	21.4 ± 1.8	23.7 ± 1.6	21.0 ± 1.1	20.9 ± 1.1	23.8 ± 1.8
Alanine aminotransferase	(U/l)	27.7 ± 3.7	26.1 ± 3.2	28.4 ± 3.1	23.4 ± 1.8	24.0 ± 2.6	26.3 ± 2.7
γ-glutamyl transpeptidase	(U/l)	48.3 ± 10.8	47.4 ± 10.6	54.8 ± 14.5	45.3 ± 8.2	42.7 ± 8.7	50.7 ± 12.2
Creatinine	(mg/dl)	0.8 ± 0.03	0.8 ± 0.06	0.8 ± 0.04	0.8 ± 0.03	0.8 ± 0.04	0.8 ± 0.04
Urea nitrogen	(mg/dl)	12.6 ± 0.4	12.1 ± 0.8	11.9 ± 0.4	12.0 ± 0.5	11.1 ± 0.5	12.0 ± 0.6
alpha-tocopherol	(μmol/l)	27.0 ± 1.5	27.6 ± 1.4	27.6 ± 1.4	26.7 ± 1.7	26.0 ± 1.6	26.3 ± 1.6

Values are means ± SE, n = 15.

†; p <0.05 vs. corn oil, paired t test.

Changes in serum lipids and lipid-related proteins in CO and FO periods are shown in Table 5. No significant differences in lipids and lipid-related proteins were observed at 0 week. In the FO period, CETP concentrations were significantly lower at 12 weeks than at 0 week and were significantly lower than in the CO period at 12 weeks. Total cholesterol, LDL-, HDL-, non HDL-cholesterol, apolipoprotein (Apo) A-1 and Apo B concentrations were significantly lower in the FO period than in the CO period after 12 weeks. TG, RLP-cholesterol, apo C-3, apo E and apo B48 concentrations didn’t change significantly during the CO and FO periods.

**Table 5:** **Changes in serum lipids and lipid-related proteins in corn oil and flaxseed oil periods**

		Corn oil period			Flaxseed oil period		
		0 week	4 week	12 week	0 week	4 week	12 week
Total cholesterol	(mg/dl)	212.2 ± 7.7	204.9 ± 7.0	216.9 ± 7.7	210.1 ± 5.8	204.8 ± 6.7	202.3 ± 7.3^†^
LDL-cholesterol	(mg/dl)	135.3 ± 7.8	127.4 ± 7.3	137.9 ± 7.7	131.2 ± 6.1	126.3 ± 6.3	127.7 ± 7.2^†^
HDL-cholesterol	(mg/dl)	51.0 ± 2.3	51.8 ± 2.0	54.9 ± 3.0	53.3 ± 3.0	53.7 ± 2.7	49.2 ± 2.7^†^
non HDL-cholesterol	(mg/dl)	161.2 ± 8.0	153.1 ± 7.0	162.0 ± 7.8	156.9 ± 6.6	151.1 ± 6.9	153.1 ± 7.0^†^
Triglyceride	(mg/dl)	139.5 ± 13.7	141.3 ± 13.9	127.0 ± 17.0	126.1 ± 11.1	134.3 ± 15.2	112.9 ± 9.1
RLP-cholesterol	(mg/dl)	5.1 ± 0.5	5.0 ± 0.4	4.8 ± 0.6	4.4 ± 0.3	5.0 ± 0.5	4.1 ± 0.2
CETP	(μg/ml)	2.0 ± 0.1	2.0 ± 0.1	1.9 ± 0.1	1.9 ± 0.1	1.9 ± 0.1	1.8 ± 0.1^*†^
apoA-1	(mg/dl)	133.7 ± 5.7	133.7 ± 5.6	136.3 ± 5.1	135.1 ± 6.0	136.5 ± 5.9	129.1 ± 6.2^†^
apoB	(mg/dl)	106.6 ± 6.0	102.7 ± 5.1	106.7 ± 5.3	105.5 ± 4.9	101.4 ± 4.8	100.5 ± 5.0^†^
apoC-3	(mg/dl)	10.5 ± 0.6	10.7 ± 0.7	10.4 ± 0.7	9.9 ± 0.5	10.2 ± 0.6	9.6 ± 0.5
apoE	(mg/dl)	4.2 ± 0.2	4.2 ± 0.2	4.3 ± 0.3	4.3 ± 0.2	4.1 ± 0.3	3.9 ± 0.2
apoB48	(mg/dl)	3.8 ± 0.5	3.7 ± 0.5	3.8 ± 0.5	3.0 ± 0.4	3.7 ± 0.5	3.6 ± 0.6

Values are means ± SE, n = 15.

RLP-cholesterol; remnant like particles-cholesterol, CETP; cholesteryl ester transfer protein.

*; p <0.05 vs. baseline, repeated-measures ANOVA with Bonferroni correction, †; p <0.05 vs. corn oil, paired t test.

Adverse events such as headache, fatigue, diarrhoea and stomach fullness were observed in both periods but were unrelated to the test diet. Moreover, no symptoms or side-effects of test diets were noted during inquiries about symptoms or during examinations.

### Changes in serum fatty acid concentrations

Changes in serum fatty acid concentrations in CO and FO periods are shown in Table 6. With the exception of docosatetraenoic acid, no significant differences in serum fatty acid composition were detected between the two periods at 0 week. At 4 weeks, LA concentrations were significantly increased in the CO period compared with 0 week. Whereas in the FO period, concentrations of LA at 4 and 12 weeks were significantly lower than in the CO period. Arachidonic acid concentrations were significantly decreased at 12 weeks in both periods and were significantly lower in the FO period than in the CO period at 4 weeks.

**Table 6:** **Changes in serum n-6 and n-3 fatty acids concentrations in corn oil and flaxseed oil periods**

		Corn oil period			Flaxseed oil period		
		0 week	4 week	12 week	0 week	4 week	12 week
Linoleic acid	(μg/ml)	907.2 ± 19.4	1004.7 ± 29.2^*^	908.8 ± 30.5	868.7 ± 23.3	909.6 ± 17.7^†^	792.0 ± 20.6^†^
γ-linolenic acid	(μg/ml)	15.9 ± 1.6	15.8 ± 2.5	12.0 ± 1.5	12.7 ± 1.4	12.1 ± 1.4	10.5 ± 1.2
Eicosadienoic acid	(μg/ml)	7.1 ± 0.3	7.0 ± 0.3	6.2 ± 0.3	6.5 ± 0.4	6.3 ± 0.4	5.3 ± 0.2^*†^
Dihomo-γ-linolenic acid	(μg/ml)	44.4 ± 2.7	40.7 ± 2.7	36.0 ± 2.8^*^	39.4 ± 2.2	33.8 ± 2.3^†^	31.2 ± 2.0^*^
Arachidonic acid	(μg/ml)	201.4 ± 10.7	187.3 ± 10.2	164.7 ± 7.6^*^	188.5 ± 10.1	172.7 ± 9.7^†^	163.5 ± 9.4^*^
Docosatetraenoic acid	(μg/ml)	5.8 ± 0.5	5.3 ± 0.4	4.1 ± 0.2^*^	4.8 ± 0.3^†^	4.5 ± 0.4^†^	3.8 ± 0.3^*^
alpha-linolenic acid	(μg/ml)	33.4 ± 2.4	30.7 ± 2.6	24.2 ± 1.8^*^	27.5 ± 2.5	92.8 ± 7.4^*†^	86.1 ± 8.7^*†^
Eicosapentaenoic acid	(μg/ml)	77.5 ± 16.1	67.9 ± 10.8	49.6 ± 7.9^*^	77.7 ± 16.6	91.3 ± 15.6^†^	71.0 ± 10.7^†^
Docosapentaenoic acid	(μg/ml)	24.9 ± 2.7	22.7 ± 2.2	17.9 ± 1.7^*^	22.5 ± 2.4	24.5 ± 1.9	19.6 ± 1.4
Docosahexaenoic acid	(μg/ml)	138.1 ± 16.5	130.8 ± 14.7	107.4 ± 12.9^*^	132.9 ± 16.3	123.3 ± 12.9	102.8 ± 9.8^*^
n-6/n-3 ratio		5.1 ± 0.5	5.6 ± 0.5	6.5 ± 0.6^*^	5.3 ± 0.6	3.8 ± 0.3^*†^	3.9 ± 0.3^*†^

Values are means ± SE, n = 15.

*; p <0.05 vs. baseline, repeated-measures ANOVA with Bonferroni correction, †; p <0.05 vs. corn oil, paired t test.

ALA concentrations were significantly decreased at 12 weeks in the CO period, but were increased at 4 and 12 weeks in the FO period compared with 0 week and were significantly higher than in the CO period at these time points.

At 4 and 12 weeks, EPA concentrations were significantly higher in the FO period than in the CO period. However, no significant differences in docosapentaenoic acid or DHA concentrations were detected between the periods, and DHA concentrations significantly decreased between 0 week and 12 weeks in both periods. Ratios of n-6/n-3 significantly increased in the CO period at 12 weeks, but were significantly decreased in the FO period at 4 and 12 weeks compared with 0 week. Moreover, n-6/n-3 ratios significantly differed between the periods at 4 and 12 weeks.

### Serum sd-LDL concentrations

**Figure 1:**
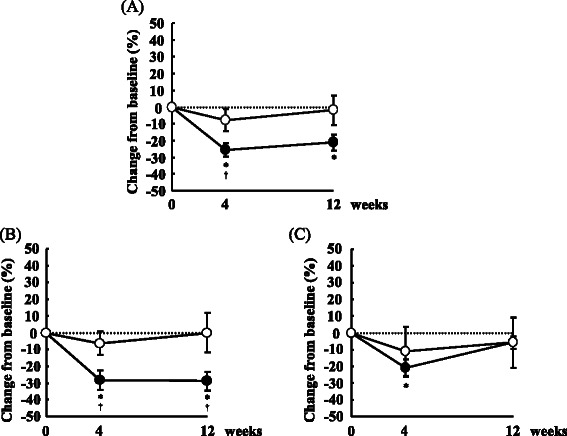
Mean (±SE) percentage changes from baseline in serum sd-LDL concentrations. 15 subjects **(A)**, 10 subjects with TG concentrations of >100 mg/dl **(B)**, 5 subjects with TG concentrations of <100 mg/dl **(C)**; open circles indicate corn oil period, and closed circles indicate flaxseed oil period; *; p <0.05 vs. baseline, repeated-measures ANOVA with Bonferroni correction, †; p <0.05 vs. corn oil, paired t test.

At 0 week, serum sd-LDL concentrations did not significantly differ between the two periods (data not shown). Changes in serum sd-LDL concentrations from 0 week are shown in Figure 1. FO supplementation was associated with 25.8% and 21.2% decreases in sd-LDL concentrations at 4 and 12 weeks, respectively, although CO supplementation did not affect sd-LDL concentrations (Figure 1A). At 4 weeks, sd-LDL concentrations were significantly lower in the FO period than in the CO period.

Previous report showed that LDL particle size was influenced by TG concentrations [4]. To elucidate the effect of baseline TG concentrations, we divided subjects into two groups according to TG concentrations. Except for TG concentrations, there were no significant differences between the TG > 100 mg/dl group and the TG < 100 mg/dl group at baseline (Table 1), although sdLDL concentrations tended to be different (p = 0.055). Among subjects with TG concentrations of >100 mg/dl, FO supplementation led to 28.2% and 28.9% reductions in sd-LDL concentrations at 4 and 12 weeks compared with 0 week, respectively, and sd-LDL concentrations significantly differed between the periods at both 4 and 12 weeks (Figure 1B). However, among subjects with TG concentration of <100 mg/dl, sd-LDL concentrations did not differ between FO or CO supplementation at any time point, but were significantly lower at 4 weeks in the FO period compared with 0 week (Figure 1C).

## Discussion

In the present study, we showed that FO supplementation markedly reduces serum sd-LDL concentrations, particularly in subjects with TG concentrations of >100 mg/dl. We also demonstrated that serum total cholesterol, LDL-cholesterol, HDL-cholesterol, CETP, Apo A-1 and Apo B concentrations were significantly lower in the FO period than in the CO period after 12 weeks.

FO used in this study is a rich source of ALA, and previous meta-analyzes suggest that ALA consumption may confer cardiovascular benefits, with a 10% decrease in risk of CHD mortality for each 1 g/d increment of ALA intake [14]. Furthermore, a recently published review showed the current evidence for an association between ALA and type 2 diabetes, and fracture risk, in addition to CVD outcomes [15].

ALA is converted in the liver into EPA and DHA [16,17], and n-6 fatty acids such as LA [18:2(n-6)] are believed to decrease the conversion of ALA to longer-chain n-3 PUFA by competing with ALA for binding to the rate-limiting enzyme Δ6-desaturase [16,18]. Other dietary factors such as polyunsaturated to saturated fat ratios, relative consumption of EPA and trans fatty acids and amounts and types of protein consumed have also been shown to affect this conversion [19]. In the present study, EPA concentrations were significantly higher after 4 and 12 weeks in the FO period than in the CO period, but DHA concentrations did not differ between the periods. Reportedly, humans convert <5% of dietary ALA to EPA or DHA [12,20]. In a previous randomised, double-blind trial, 56 participants were given 3-g doses of ALA/d from FO or olive oil placebo capsules. After 12 weeks, plasma EPA and docosapentaenoic acid (DPA) concentrations were significantly increased among subjects taking FO, whereas plasma DHA concentrations did not change [21]. Thus, the present increases in serum EPA concentrations may be clinically relevant. However, in agreement with previous studies [22,23], increased ALA intake failed to increase plasma phospholipid DHA concentrations, suggesting that the conversion to DHA is extremely low.

Previous study showed that non HDL-cholesterol (LDL + IDL + VLDL cholesterol) was more potent predictors of CVD [24]. In this study, non HDL-cholesterol was significantly lower in the FO period than in the CO period after 12 weeks. Therefore, it indicates that the FO supplementation could contribute to the improvement of the lipid profile. On the other hand, HDL-cholesterol concentrations were also significantly lower in the FO period than in the CO period in this study. Other studies also showed that HDL-cholesterol concentrations were lower in the ALA group than in the LA group [25,26]. In another study, no significant differences in HDL-cholesterol concentrations were found compared with the ALA group and the LA group [27]. Therefore, the effect of ALA on HDL-cholesterol concentrations is not consistent.

Increased hepatic production and/or retarded clearance from plasma of large VLDL result in increased production of precursors of sdLDL particles. Thus far, seven distinct LDL subspecies, which differ in their metabolic behavior and pathological rules, have been identified [28]. Plasma VLDL concentrations correlate with increased density and decreased sizes of LDLs [29,30]. In addition, LDL size and density are inversely correlated with plasma concentrations of HDLs, particularly those of the HDL2 subclass [31]. Sd-LDL particles are produced from intravascular processing of specific larger VLDL precursors through a series of steps, including lipolysis [28]. A common lipoprotein profile, designated the atherogenic lipoprotein phenotype, is characterised by a predominance of sd-LDL particles. Multiple features of this phenotype, including increased concentrations of triglyceride rich lipoprotein remnants and IDLs, and reduced concentrations of HDL and associated insulin resistance, contribute to the risk of coronary heart disease, particularly compared with individuals with predominantly larger LDLs. Reportedly, sd-LDL uptake by arterial tissue is greater than for larger LDLs [32], suggesting greater transendothelial transport of smaller particles. In addition, smaller LDL particles may have decreased receptor-mediated uptake and increased proteoglycan binding [33,34]. Several in vitro studies have demonstrated that LDL subfractions differ in their susceptibility to oxidative stress, which is a significant atherogenic factor [35,36]. Thus, increased concentrations of sd-LDL subfractions may significantly contribute to cardiovascular risk [37,38]. In the present study, sd-LDL concentrations were markedly reduced in the FO period, particularly in subjects with TG concentrations of >100 mg/dl. Thus, ALA in FO may lower sd-LDL cholesterols and contribute to reduce cardiovascular disease risk. Furthermore, although TG normal levels are defined as <150 mg/dl, previous study showed that the TG cutoff point that best distinguishes the two phenotypes, which are characterized by a predominance of large, buoyant LDL particles and sd-LDL particles, was 95 mg/dl in primarily healthy study sample [4]. Therefore, we considered that the changes of serum sd-LDL concentrations, particularly in subjects with TG concentrations of >100 mg/dl could be observed. However, we must take into account the small subgroup sample size in this study. Studying more subjects is clearly needed to determine the effect of TG concentrations on the FO supplementation.

Previously, it was reported that n-3 fatty acids, such as ALA, EPA and DHA, reduced secretion of apo B, induced degradation of apo B [39,40] and decreased hepatic VLDL production [39]. There are two types of apo B (apo B100 and apo B48). Apo B100 is produced in the liver and apo B48 is produced in the proximal small intestine. In this study, we analyzed apo B and apo B48 concentrations. Apo B concentrations were significantly lower in the FO period than in the CO period after 12 weeks, although apo B48 concentrations didn’t change significantly during the CO and FO periods. These results suggest that apo B, especially apo B100 concentrations, were significantly lower in the FO period. Furthermore, a previous report showed that degradation of CETP activity after EPA treatments may contribute to lower sd-LDL concentrations [41]. In this study, serum ALA and EPA concentrations were significantly increased in the FO period compared with the CO period, and CETP and Apo B concentrations were significantly lower in the FO period than in the CO period. These data suggest that FO-mediated decreases in sd-LDL concentrations may reflect decreases in apo B and VLDL concentrations. A limitation of our study is that we didn’t analyze the discrimination of HDL2/HDL3-subclasses. Previous report suggested that LDL size and density are inversely correlated with plasma concentrations of HDLs, particularly those of the HDL2 subclass [31]. Therefore, it is necessary to analyze the discrimination of HDL2/HDL3-subclasses for further study.

This study has shown that FO supplementation markedly decreases serum sd-LDL concentrations, particularly in subjects with TG concentrations of >100 mg/dl. In conclusion, this study indicates that the FO, which is a rich source of ALA, leads to lower sd-LDL cholesterol concentrations.

## Abbreviations

ALA: Alpha-linolenic acid
FO: Flaxseed oil
sd-LDL: Small dense low-density lipoprotein
CO: Corn oil
TG: Triglyceride
HDL: High-density lipoprotein
CVD: Cardiovascular disease
EPA: Eicosapentaenoic acid
DHA: Docosahexaenoic acid
CETP: Cholesteryl ester transfer protein
Apo: Apolipoprotein
